# Influence of Network Topology on the Viscoelastic Properties of Dynamically Crosslinked Hydrogels

**DOI:** 10.3389/fchem.2020.00536

**Published:** 2020-06-30

**Authors:** Emilia M. Grad, Isabell Tunn, Dion Voerman, Alberto S. de Léon, Roel Hammink, Kerstin G. Blank

**Affiliations:** ^1^Mechano(bio) Chemistry, Max Planck Institute of Colloids and Interfaces, Potsdam, Germany; ^2^Department of Molecular Materials, Institute for Molecules and Materials, Radboud University, Nijmegen, Netherlands; ^3^Department of Tumor Immunology, Radboud Institute for Molecular Life Sciences, Radboud University Medical Center, Nijmegen, Netherlands; ^4^Division of Immunotherapy, Oncode Institute, Radboud University Medical Center, Nijmegen, Netherlands

**Keywords:** hydrogel, rheology, coiled coil, polyisocyanopeptide, polyethylene glycol, relaxation time, network topology, multivalency

## Abstract

Biological materials combine stress relaxation and self-healing with non-linear stress-strain responses. These characteristic features are a direct result of hierarchical self-assembly, which often results in fiber-like architectures. Even though structural knowledge is rapidly increasing, it has remained a challenge to establish relationships between microscopic and macroscopic structure and function. Here, we focus on understanding how network topology determines the viscoelastic properties, i.e., stress relaxation, of biomimetic hydrogels. We have dynamically crosslinked two different synthetic polymers with one and the same crosslink. The first polymer, a polyisocyanopeptide (PIC), self-assembles into semi-flexible, fiber-like bundles, and thus displays stress-stiffening, similar to many biopolymer networks. The second polymer, 4-arm poly(ethylene glycol) (starPEG), serves as a reference network with well-characterized structural and viscoelastic properties. Using one and the same coiled coil crosslink allows us to decouple the effects of crosslink kinetics and network topology on the stress relaxation behavior of the resulting hydrogel networks. We show that the fiber-containing PIC network displays a relaxation time approximately two orders of magnitude slower than the starPEG network. This reveals that crosslink kinetics is not the only determinant for stress relaxation. Instead, we propose that the different network topologies determine the ability of elastically active network chains to relax stress. In the starPEG network, each elastically active chain contains exactly one crosslink. In the absence of entanglements, crosslink dissociation thus relaxes the entire chain. In contrast, each polymer is crosslinked to the fiber bundle in multiple positions in the PIC hydrogel. The dissociation of a single crosslink is thus not sufficient for chain relaxation. This suggests that tuning the number of crosslinks per elastically active chain in combination with crosslink kinetics is a powerful design principle for tuning stress relaxation in polymeric materials. The presence of a higher number of crosslinks per elastically active chain thus yields materials with a slow macroscopic relaxation time but fast dynamics at the microscopic level. Using this principle for the design of synthetic cell culture matrices will yield materials with excellent long-term stability combined with the ability to locally reorganize, thus facilitating cell motility, spreading, and growth.

## Introduction

Biological materials are increasingly serving as inspiration for the synthesis of smart and sustainable polymeric materials, both in engineering and biomedical application areas. Integrating the desired mechanical performance with (multi-)functionality, e.g., stimuli-responsiveness and self-healing, requires a detailed understanding of how molecular structure translates into material architecture and function. Key features of biological materials are their hierarchical structure, built up via the well-defined self-assembly of molecular building blocks (Kushner and Guan, 2011; Egan et al., 2015), as well as their viscoelastic behavior (Kollmannsberger and Fabry, 2011; Gralka and Kroy, 2015) combined with non-linear stress-strain responses (Storm et al., 2005; Kollmannsberger and Fabry, 2011; Gralka and Kroy, 2015). Focusing on materials with biomedical relevance, the cytoskeleton (actin, intermediate filaments, and microtubules) and the extracellular matrix (ECM; e.g., collagen and fibrin) of mammalian cells are well-studied examples of biological hydrogel networks that combine these properties (Storm et al., 2005; Kollmannsberger and Fabry, 2011; Gralka and Kroy, 2015). Protein building blocks of the cytoskeleton and the ECM self-assemble into semi-flexible fiber bundles. The largely entropic response of these bundles to stretching forces causes the network to become stiffer with increasing deformation (stress-stiffening) (Storm et al., 2005). At the same time, these networks contain non-covalent crosslinks (Claessens et al., 2006; Schmoller et al., 2008, 2009; Lin et al., 2010; Lieleg et al., 2011; Lansky et al., 2015). These dynamic crosslinks dissociate and re-associate and are thus responsible for stress relaxation and self-healing.

Understanding the interplay between the above-mentioned characteristics is key for determining the mechanical properties of cells and tissues as well as for the development of synthetic cell culture matrices that mimic the natural ECM. For example, it has been shown that both stress-stiffening (Das et al., 2016) and stress relaxation (McKinnon et al., 2014; Chaudhuri et al., 2015, 2016; Tang et al., 2018) are key factors affecting cell spreading and stem cell differentiation. It has remained a significant challenge, however, to systematically vary these parameters and to establish relationships between network topology and linear as well as non-linear viscoelastic properties. Considering natural biopolymer networks, the majority of studies have focused on reconstituted actin networks, crosslinked with different natural crosslinking proteins (Claessens et al., 2006; Schmoller et al., 2008, 2009; Lieleg et al., 2011) or synthetic crosslinking modules (Lorenz et al., 2018). These studies have shown that the crosslink properties (kinetics and stiffness) affect network topology, elastic modulus as well as stress relaxation and aging (Claessens et al., 2006; Schmoller et al., 2008, 2009; Lieleg et al., 2011; Strehle et al., 2011; Wei et al., 2016). Analogous studies aimed at understanding the effect of crosslink kinetics on viscoelastic material responses have been performed for a number of synthetic polymeric materials. Besides investigating the effect of the crosslink properties themselves (Yount et al., 2005; Shen et al., 2007; Appel et al., 2014; Rossow et al., 2014; Grindy et al., 2015; Tunn et al., 2018), these studies have focused on the contributions of network defects such as dangling ends and loops (Annable et al., 1993; Rossow et al., 2014; Ciarella et al., 2018), crosslink functionality (Li et al., 2016; Gu et al., 2018; Tunn et al., 2019), and polymer length (Annable et al., 1993; Tan et al., 2017). Even though a direct comparison is difficult due to the different polymers used, it can generally be concluded that the number of elastically active chains and their ability to relax after crosslink dissociation are key parameters that determine the macroscopic relaxation time of a material.

**Figure 1:**
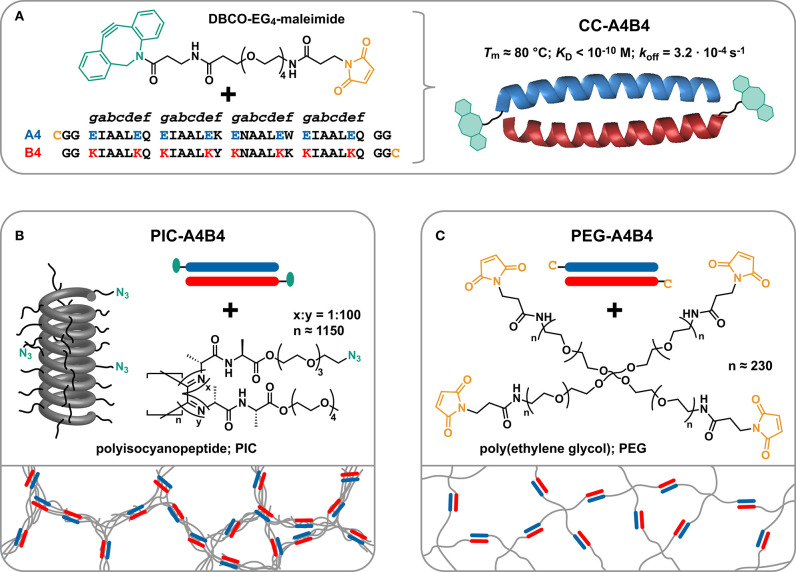
Experimental design. **(A)** Functional groups utilized for coupling the coiled coils (CCs) to polyisocyanopeptide (PIC) and poly(ethylene glycol) (PEG) polymers. The CC-forming peptides A4 and B4 each carry a terminal cysteine (Cys; C) introduced during solid-phase peptide synthesis. The Cys is reacted with the heterobifunctional linker DBCO-EG_4_-maleimide. **(B)** Synthesis of CC-crosslinked polyisocyanopeptide hydrogels (PIC-A4B4). The DBCO-functionalized CCs are reacted with the PIC polymers that carry azide groups with an average spacing of 11.5 nm. **(C)** Synthesis of CC-crosslinked poly(ethylene glycol) hydrogels (PEG-A4B4). The Cys-terminated CCs are directly reacted with maleimide-functionalized 4-arm PEG (starPEG; MW = 40 kDa).

With the goal of gaining more detailed insights into how network topology affects stress relaxation of hydrogels, we have crosslinked two synthetic polymer networks possessing fundamentally different network architecture with one and the same crosslink and compared the stress relaxation behavior of the resulting networks. As the crosslink, we used a synthetic coiled coil (CC; Figure 1). CCs are self-assembled superhelical structures (Lupas, 1996; Woolfson, 2005) that occur naturally within many cytoskeleton and ECM proteins, where they are either part of the fibers themselves (e.g., intermediate filaments and fibrin) or are structural components of actin crosslinking proteins (e.g., myosin and α-actinin). CCs possess a so-called heptad repeat sequence, termed *abgdefg* (Figure 1). Hydrophobic amino acids usually occupy the *a* and *d* positions. In the folded superhelical structure, the hydrophobic side chains align on one face of the helix and constitute a hydrophobic core. The *e* and *g* positions are frequently filled with charged amino acids, which play an important role in defining helix orientation and oligomerization specificity. The solvent-exposed amino acids *b, c*, and *f* are more variable, while their helix propensity is an important factor contributing to overall CC stability. Synthetic CCs of controlled length and sequence have evolved into tunable protein-based building blocks for synthetic biology and materials science where they find application in protein origami structures (Fletcher et al., 2013; Ljubetič et al., 2017) and as crosslinks for polymeric materials (Petka et al., 1998; Wang et al., 1999; Yang et al., 2006; Shen et al., 2007; Dånmark et al., 2016; Tunn et al., 2018, 2019). Based on their natural abundance in biological materials and their generally established application as molecular building blocks, we consider CCs to be excellent tunable crosslinks for biomimetic material design.

One network to be crosslinked with these CC building blocks consists of water-soluble, semi-flexible polyisocyanopeptides (PICs) (Cornelissen et al., 2001; Kouwer et al., 2013). PICs are fully synthetic, helical polymers, known to self-assemble into fiber-like architectures with stress-stiffening properties. Each monomer is functionalized with a dialanyl peptide, which introduces a hydrogen bond network parallel to the helical axis. These hydrogen bonds stabilize the helical structure and also contribute to the stiffness of these polymers (van Buul et al., 2013) with persistence lengths *L*_p_ >10 nm (Jaspers et al., 2016; Kouwer et al., 2018; Schoenmakers et al., 2018a). Each monomer further carries oligo(ethylene glycol) units that cause a phase transition when increasing the temperature above the lower critical solution temperature (LCST). Upon heating, the polymers become hydrophobic and start to bundle, thereby forming a physically crosslinked hydrogel network at very low concentrations (Kouwer et al., 2013; Jaspers et al., 2014; Vandaele et al., 2020). Stress-stiffening PIC networks thus have the potential to serve as synthetic mimics of the cytoskeleton (Jaspers et al., 2017) and the ECM (Das et al., 2016), both for fundamental studies and for cell culture applications.

To fully utilize the potential of PIC hydrogels as cytoskeleton and ECM mimics, several types of crosslinks have previously been introduced into PIC networks. These include short double-stranded DNA oligonucleotides (Deshpande et al., 2016) and stimuli-responsive DNA motifs (Deshpande et al., 2017) as well as self-assembled virus capsids (Schoenmakers et al., 2018b) and covalent triazole crosslinks (Schoenmakers et al., 2018a). In the majority of these studies, the focus was placed on understanding the effect of these crosslinks on the non-linear stress-stiffening response. The CC-crosslinked PIC hydrogel (PIC-A4B4; Figure 1) developed here is utilized for investigating the relationship between the PIC network properties and stress relaxation. Most importantly, we compare the viscoelastic properties of these CC-crosslinked PIC networks with a well-characterized reference network, based on terminally crosslinked 4-arm poly(ethylene glycol) (PEG-A4B4; Figure 1; Sakai et al., 2008; Lange et al., 2011; Asai et al., 2012; Akagi et al., 2013; Grindy et al., 2015; Tunn et al., 2018). Our results show that the relaxation time assigned to CC dissociation varies between the two networks and is longer for the PIC-A4B4 network. This demonstrates that the macroscopic network relaxation time is not determined by the kinetics of the crosslinks alone. It also depends on the network topology and thus the ability of elastically active network chains to relax after crosslink dissociation. We attribute the slower relaxation of PIC-A4B4 networks to the close proximity of multiple crosslinks along the same elastically active chain, so that the dissociation of one crosslink does not necessarily relax the entire chain. Such multivalency effects thus have to be considered when designing polymeric networks with a controlled relaxation time.

## Materials and Methods

### Synthesis and Characterization of Azide-Functionalized PIC Polymers

The isocyanopeptide monomers IC-AA-(EG)_4_-OMe and IC-AA-(EG)_3_-N_3_ were synthesized according to Mandal et al. (2013). For the synthesis of azide-functionalized polymers, azide- and methoxy-terminated monomers were mixed in a 1:100 ratio (total concentration of 50 mg ml^−1^ in toluene). The (Ni(ClO_4_)_2_)∙6H_2_O catalyst was dissolved in a 9:1 mixture of toluene and absolute ethanol. The pre-dissolved catalyst was added to the monomer mixture in a catalyst:monomer ratio of 1:10,000. The mixture was stirred for 2–3 days and precipitated in di-isopropyl ether (3x), resulting in an off-white solid. The polymer was analyzed with viscometry as described (Mandal et al., 2013). The average molecular weight of the polymer as determined from its viscosity is 412 kg mol^−1^.

### Synthesis of A4B4-Crosslinked PIC Hydrogels

The CC-forming peptides A4 and B4 were obtained from Pepscan (Lelystad, The Netherlands) and Proteogenix (Schiltigheim, France) in a purity >95% and with trifluoroacetic acid (TFA) counter ions. For bioconjugation, the A4 peptide carries a cysteine residue at its N-terminus while the cysteine is located at the C-terminus for B4. For both peptides, the N-terminus was amidated and the C-terminus was acetylated. Equimolar amounts of A4 and B4 (1 mM each) were dissolved in phosphate buffered saline (PBS; 10 mM Na_2_HPO_4_/1.8 mM KH_2_PO_4_ pH 7.4, 137 mM NaCl, 2.7 mM KCl) to allow CC formation. The heterobifunctional crosslinker DBCO-PEG_4_-maleimide (DBCO: dibenzocyclooctyne; Jena Bioscience, Jena, Germany) was dissolved in DMSO to a concentration of 100 mM. It was added to the CC-A4B4 solution to a final concentration of 2 mM to yield a 1:1 Cys:maleimide ratio. The solution was incubated for 1 h at 4°C and 700 rpm. Functionalization of CC-A4B4 with the crosslinker was confirmed with MALDI-TOF. A desalted sample was mixed with the matrix α-cyano-4-hydroxycinnamic acid and analyzed in linear-positive mode (Supplementary Figure 1).

For hydrogel synthesis, the azide-functionalized PIC polymer was dissolved to a concentration of 2.5 mg ml^−1^ in PBS while incubating the sample at 4°C for 48 h. The polymer solution was mixed with the DBCO-functionalized CC-A4B4 to obtain a 1:1 ratio of azide:DBCO (0.185 mM each; 2 mg ml^−1^ PIC). During this preparation process, all components and the freshly prepared mixture were kept on ice. The mixture was immediately loaded onto the rheometer (plate pre-cooled to 7°C) and gelation was allowed to occur in the rheometer after the gap size was adjusted.

### Synthesis of A4B4-Crosslinked PEG Hydrogels

The CC-forming peptides A4 and B4 were each dissolved to a concentration of 4 mM in PBS. Star-shaped 4-arm poly(ethylene glycol) (starPEG) with terminal maleimide groups (40 kDa, polydispersity index = 1.02; JenKem Technology USA, Plano, TX, USA) was dissolved to a concentration of 1 mM in PBS. The peptides A4 and B4 were mixed in a 1:1 ratio (25 μl each) to allow for CC formation. Immediately after, the starPEG solution was added to obtain a 1:1 Cys:maleimide ratio (50 μl). This yields a final concentration of 0.5 mM starPEG and a total peptide concentration of 2 mM. The reaction mixture was incubated for 15 min at 800 rpm at room temperature. After this incubation time, the sample was thoroughly mixed by pipetting up and down several times to form a homogeneous PEG-A4B4 hydrogel. In order to remove entrapped air bubbles, the hydrogel was centrifuged for 2 min at 2,000 g.

### Rheology of PIC Hydrogels

All measurements were performed with a stress-controlled rheometer (MCR-302, Anton Paar, Ostfildern, Germany), using parallel-plate geometry (diameter 25 mm, stainless steel). The initial gap was adjusted to 200 μm, while controlling the normal force (0 N ± 0.1 N). Silicone oil (Sigma-Aldrich 378364, viscosity 100 cSt @ 25°C) was used to prevent sample evaporation. In general, CC-A4B4 crosslinked PIC samples (PIC-A4B4) were subjected to different temperature protocols: (1) 7°C → 55°C (rate = 1°C min^−1^), T = 55°C constant for 90 min; (2) 7°C → 55°C (rate = 1°C min^−1^), T = 55°C constant for 90 min, 55°C → 20°C (rate = 1°C min^−1^), T = 20°C constant for ≥10 min; (3) 7°C → 20°C (rate = 1°C min^−1^), T = 20°C constant for 10 h. In addition, a PIC sample without CC-A4B4 crosslinks (PIC-0) was subjected to the same protocols.

The linear viscoelastic properties (storage modulus *G*′ and loss modulus *G*″) were recorded during each respective temperature protocol. The measurements were carried out at a strain amplitude γ of 1% and a frequency *f* of 1.6 s^−1^ (angular frequency ω = 10 rad s^−1^). The temperature protocol was followed by either an amplitude or frequency sweep. For the amplitude sweeps, *f* was set to 1.6 s^−1^ while γ was varied from 1 to 1,000%. For the frequency sweeps, γ was 1% and *f* was varied from 10 to 0.0001 s^−1^. Each experiment was performed in triplicate. One data set is shown in the main text while the two additional data sets are presented in the Supplementary Material.

To investigate the non-linear viscoelastic properties of the different samples, a pre-stress protocol was performed as originally introduced and validated for dynamically crosslinked biopolymer networks by Broedersz et al. (2010). It was subsequently adapted to PIC hydrogels by Kouwer et al. (2013). Each pre-stress experiment directly followed one of the different temperature protocols (1, 2, or 3). As part of the pre-stress protocol, the samples were subjected to a constant pre-stress (σ) in the range from 0.5 to 600 Pa while a small oscillatory stress was applied in addition (δσ). In all cases, the amplitude of the oscillatory stress was <10% of the pre-set constant pre-stress (see Supplementary Material for details). At a given pre-stress, a frequency sweep was performed (0.1–10 s^−1^) to validate that the material response is frequency independent. Subsequently, the resulting oscillatory strain (δγ) was determined at a frequency of 1 s^−1^. The differential modulus *K*′ (δσ/δγ) was determined from the applied oscillatory stress (δσ) and the measured strain (δγ) values. The resulting *K*′ values were normalized to the plateau modulus *G*_0_, which was obtained from averaging the storage modulus *G*′ measured at the pre-stress values of 1, 1.2, and 1.5 Pa (linear viscoelastic range). *K*′/*G*_0_ was plotted against the applied constant pre-stress σ. The critical stress (σ_c_) was obtained from this plot and corresponds to the value of σ where *K*′ is not constant anymore. Each experiment was performed in duplicate or triplicate. One data set is shown in the main text while the additional data sets are presented in the Supplementary Material.

### Rheology of PEG Hydrogels

The PEG-A4B4 hydrogel was characterized using a 12 mm cone-plate geometry (gap 20 μm, stainless steel). First, an amplitude sweep was performed at 20°C. The strain amplitude γ was varied from 1 to 1,000% at a constant frequency *f* of 1.6 s^−1^. Second, frequency sweeps were performed using γ = 10% while *f* ranged from 15.9 to 0.0006 s^−1^. Frequency sweeps were carried out at different temperatures (20–55°C in steps of 5°C). A new sample was used for every frequency sweep. The experiment at 55°C was performed in triplicate. One data set is shown in the main text while the two additional data sets are presented in the Supplementary Material. To validate that the viscoelastic properties of the hydrogels are not affected by the measurement geometry, a control experiment was further performed with a 12 mm plate-plate geometry (gap 200 μm; see Supplementary Material).

### Detection of Hydrophobic Bundling With Nile Red

The binding of Nile Red to hydrophobic PIC bundles was determined for PIC-0 and PIC-A4B4. Nile Red (Thermo Fisher Scientific) was dissolved in DMSO to a concentration of 1 mg ml^−1^. For each hydrogel, two samples were prepared in PBS as described above. Nile Red was added to one sample (final concentration 10 μg ml^−1^) while the second sample served as a reference. Subsequently, all samples were incubated over night at ~4°C to allow complete mixing of dye and polymer. The pre-incubated samples where then subjected to a heating protocol while measuring the fluorescence intensity of Nile Red. The measurement was performed in a temperature-controlled microplate reader (Cytation5, BioTek Instruments, Inc., Winooski, VT, USA) using glass-bottom 96-well plates (SensoPlate, Greiner Bio-One, Frickenhausen, Germany). The sample was initially kept at 30°C and subsequently heated to 55°C. Following 45 min incubation at 55°C, the sample was again cooled down to 30°C. At each temperature, the fluorescence intensity was recorded. The samples were excited at 540 nm (15 nm slit width) and fluorescence emission was measured from 580 to 700 nm (15 nm slit width). Each sample was measured in triplicate and the average was taken for data analysis. The measured intensity of the reference samples was subtracted from the values measured for the Nile Red containing samples. The intensity at 655 nm (emission maximum of Nile Red) was used to compare Nile Red fluorescence at different temperatures. For this comparison, the intensity was further normalized to the initial intensity measured at 30°C.

## Results and Discussion

### Design and Synthesis of Coiled Coil-Crosslinked PIC and PEG Networks

With the goal of investigating the influence of network topology on the relaxation time of dynamically crosslinked hydrogel networks, PIC and starPEG were crosslinked with a well-characterized CC to form the hydrogels PIC-A4B4 and PEG-A4B4 (Figure 1). The CC-forming peptides A4 and B4 self-assemble into a parallel 4-heptad heterodimer with high thermodynamic and kinetic stability (Figure 1A). The melting temperature *T*_m_ and equilibrium dissociation constant *K*_D_ have been obtained from thermal unfolding experiments performed with circular dichroism spectroscopy. These experiments yielded *T*_m_ = 81°C and *K*_D_ <1.0 · 10^−10^ M at 20°C (Thomas et al., 2013). *T*_m_ remains unaffected upon conjugation to PEG (Goktas et al., 2018). Using atomic force microscopy-based single-molecule force spectroscopy, the dissociation rate *k*_off_ was determined to be 3.2 · 10^−4^ s^−1^ at 25°C (Goktas et al., 2018). The individual CC-forming peptides are not folded, while the CC itself is a highly rigid superhelix (Wolgemuth and Sun, 2006). The CC was functionalized with a cysteine residue at the N-terminus of A4 and at the C-terminus of B4 to allow covalent coupling to PIC and starPEG polymers (Figure 1).

For the synthesis of PIC hydrogels, tetra(ethylene glycol) functionalized monomers were used. These polymers possess a gelation temperature (*T*_gel_ = LCST) of ~39°C in PBS (Deshpande et al., 2016), which is well below the *T*_m_ of CC-A4B4. A fraction of monomers (1:100) was equipped with a terminal azide functional group to allow peptide coupling via a heterobifunctional DBCO-EG_4_-maleimide crosslinker (Figure 1B). The average spacing between azide functional groups can be calculated based on the known structure of polyisocyanide polymers, which is a 4_1_ helix with a pitch of 0.46 nm (Cornelissen et al., 2001). The axial distance between two monomers is thus 0.115 nm. Considering that 1% of monomers carry an azide functional group, this results in an average azide spacing of ~11.5 nm. The azide-functionalized polymer is termed PIC-0 and serves as a control for all rheology experiments.

For the synthesis of CC-crosslinked PIC-A4B4 hydrogels, an equimolar amount of the CC-forming peptides was mixed to allow CC formation. The folded CC was subsequently reacted with DBCO-EG_4_-maleimide in a 1:1 thiol:maleimide ratio. The yield of this reaction was estimated to be 90% using MALDI-TOF (Supplementary Figure 1). Subsequently, the DBCO-functionalized CC was added to the azide-containing PIC to allow CC-mediated crosslinking via a strain-promoted azide-alkyne cycloaddition reaction (N_3_:DBCO = 1:1). The yield of this reaction was previously quantified for a similar system (PIC crosslinked with DNA) and was determined to be 90% (Deshpande et al., 2016). Considering the yield of both reactions, it can be assumed that ~80% of CC-forming peptides are coupled to the PIC polymer. This ultimately results in an average spacing of CC-forming peptides of ~14 nm, which is similar to the *L*_p_ of closely related tri(ethylene glycol) functionalized PIC polymers (12–30 nm) (Jaspers et al., 2016; Kouwer et al., 2018; Schoenmakers et al., 2018a). It can thus be assumed that the *L*_p_ and the distance between crosslinks is on a similar length scale. The progress of the conjugation reaction was further followed with rheology, recording the evolution of the storage modulus *G*′ as a function of time. Performing this experiment below the LCST ensures that the crosslinking of individual polymers is monitored and that hydrophobically stabilized bundles are absent. The observed increase in *G*′ indicates that a network is indeed formed as a result of the CC-mediated crosslinking reaction (Supplementary Figure 2).

Maleimide-functionalized starPEG was used for the synthesis of the terminally crosslinked starPEG reference network (PEG-A4B4). This allowed for the direct conjugation of thiol-containing CCs to the maleimide-functionalized starPEG without the need of the heterobifunctional DBCO-PEG_4_-maleimide crosslinker (Figure 1C). For crosslinking the starPEG network, we also employed pre-assembled CCs to utilize the same synthetic strategy as used for the PIC-A4B4 networks. The starPEG concentration used was close to the critical overlap concentration where the resulting hydrogels possess a very small number of entanglements (Asai et al., 2012; Akagi et al., 2013). In terminally crosslinked starPEG hydrogels, the distance between crosslinks is defined by the size of each PEG chain, characterized by a polydispersity index of 1.02. This is a key difference to the PIC-A4B4 networks, where the distance between crosslinks is distributed around an average value that is determined by the density of azide functional groups and the yield of the coupling reaction.

### Bundle Formation and Network Topology of Coiled Coil-Crosslinked PIC Networks

Before comparing the stress relaxation behavior of PIC-A4B4 and PEG-A4B4 hydrogels, we first investigated the contributions of CC-crosslinking and hydrophobic bundling to the overall network properties of PIC-A4B4. We mixed DBCO-functionalized CC-A4B4 and PIC, transferred the sample to the rheometer, immediately heated from 7 to 55°C and incubated the sample at 55°C while recording the storage modulus *G*′. Upon heating, CC-crosslinking and bundle formation are expected to occur simultaneously. The sample was subsequently re-cooled to 20°C and its properties were compared to a control sample without CC crosslinks (PIC-0). In addition, the properties of re-cooled PIC-A4B4 were compared to a sample never heated to 55°C but incubated at 20°C for an extended period of time (10 h) (Supplementary Figure 2).

**Figure 2:**
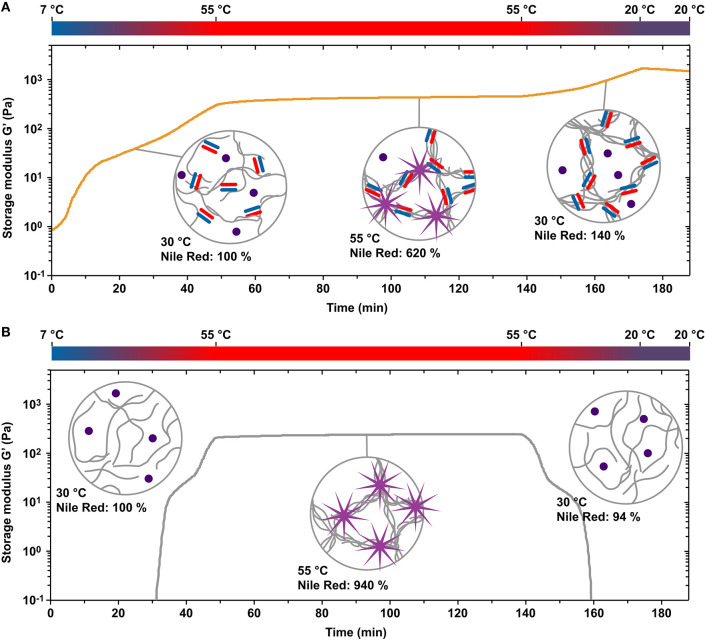
Temperature-induced hydrophobic bundle formation and gelation of PIC-A4B4 and PIC-0 hydrogels. **(A)** Evolution of the storage modulus *G*′ as a function of time when subjecting PIC-A4B4 to temperature protocol 2 (7°C → 55°C → 20°C). **(B)** Evolution of the storage modulus *G*′ as a function of time when subjecting PIC-0 to temperature protocol 2. The measurements were performed with a strain amplitude of 1% and a frequency of 1.6 s^−1^. Hydrophobic bundle formation was visualized in a temperature-controlled microplate reader using the fluorophore Nile Red, which is known to increase in intensity in hydrophobic environments. The intensity (measured at λ_ex_ = 540 nm and λ_em_ = 655 nm) was normalized to the state of the sample at a temperature of 30°C during the initial heating step.

When gradually heating the PIC-A4B4 and PIC-0 samples from 7 to 55°C, *G*′ was higher for the PIC-A4B4 samples already at the start of the recording (Figure 2). This suggests that first CC crosslinks have already formed between individual polymers while the sample was maintained at 7°C. Upon heating, *G*′ increased and hydrophobic bundles appeared in both samples, as visualized using the fluorophore Nile Red (Supplementary Table 1). Nile Red responds to hydrophobic environments with enhanced fluorescence emission and is thus a versatile reporter for bundle formation. The LCST transition appears to be broader for PIC-A4B4 than for PIC-0, suggesting that CC-crosslinks may interfere with the tight packing of PIC polymers in the bundles. This is confirmed when comparing the increase in Nile Red fluorescence for the PIC-A4B4 and PIC-0 samples. While Nile Red fluorescence increased almost 10-fold for PIC-0 only a 6-fold increase was observed for PIC-A4B4. When maintaining the samples at a constant temperature of 55°C, a plateau in *G*′ was reached. The *G*′ plateau was higher for PIC-A4B4 (440–460 Pa; Figure 2A) than for PIC-0 (240-250 Pa; Figure 2B). This provides first evidence that CC crosslinks are present in hydrophobically bundled PIC networks at 55°C and that these crosslinks contribute to the overall viscoelastic properties of these networks. When re-cooling both samples below the LCST, Nile Red fluorescence decreased to the starting value. This clearly shows that no hydrophobic bundles remain at temperatures below the LCST. At 20°C, the PIC-0 sample was a solution with very low *G*′. In contrast, *G*′ increased upon cooling for the PIC-A4B4 sample (Figure 2).

This increase in *G*′ is unexpected and has further not been observed for any other crosslinked PIC network (Deshpande et al., 2016, 2017; Schoenmakers et al., 2018a,b). As the *L*_p_ of individual PIC polymers decreases with lowering the temperature, a decrease in *G*′ is expected for bundled PIC networks as long as the network structure is not altered (Kouwer et al., 2013). One possible explanation for the observed increase in *G*′ is that the thermodynamic stability of the CC crosslinks increases while the sample is being cooled. Additional CC crosslinks thus form and stabilize the bundles. This does not explain the behavior of the PIC-A4B4 network at temperatures below the LCST, however, where the bundled network structure is no longer held together via hydrophobic interactions. We propose that the presence of CC crosslinks kinetically traps the polymers in the bundled state even though the polymers have become hydrophilic. At temperatures below the LCST, *k*_off_ of the CC crosslinks is low (t_1/2_ = 35 min at 25°C) so that the trapped bundle structures remain for extended periods of time and reorganize only slowly. This is supported by the evolution of *G*′ during repeated heating-cooling cycles (Supplementary Figure 3). Once the re-cooled sample is kept at 20°C, *G*′ starts to decrease continuously. Moreover, *G*′ decreases faster when a new heating cycle is started (i.e., *k*_off_ increases with temperature). Once the LCST is reached, hydrophobic bundles appear again and *G*′ starts to increase. It is likely that prolonged incubation at 20°C yields the same final network structure and viscoelastic properties as a sample that was never subjected to any heating-cooling cycle (Supplementary Figure 2). It should be noted that internal stress may accumulate in trapped bundle structures, which may further contribute to the increase in *G*′ upon cooling. Internal stress is also relaxed via crosslink dissociation as has been observed for actin networks polymerized in the presence of the crosslinking protein fascin (Lieleg et al., 2011).

At this moment, we can only speculate about the structure of the network equilibrated at 20°C. Considering the properties of individual polymers, the estimated distance between CCs is ~14 nm. This is on the same length scale as the *L*_p_ determined for closely related tri(ethylene glycol) functionalized PIC polymers (12–30 nm) (Jaspers et al., 2016; Kouwer et al., 2018; Schoenmakers et al., 2018a). As a result of their increased bulkiness, we expect *L*_p_ to be larger for our tetra(ethylene glycol) functionalized polymers. The average distance between crosslinks is thus expected to be at least similar to—and possibly shorter than—the *L*_p_ of individual PIC polymers. Crosslinking may thus cause the partial alignment of PIC polymers into bundle-like structures even at temperatures below the LCST. When comparing *G*′ of the PIC-A4B4 networks, *G*′ is clearly lower for networks equilibrated at 20°C (62 Pa after 10 h of incubation; Supplementary Figure 2) than for hydrophobically bundled networks at 55°C (440–460 Pa; Figure 2A). CC-crosslinking alone is thus not sufficient to obtain a fully bundled network. The network structure is most likely heterogeneous and consists of a mixture of CC-crosslinked bundles and individual polymers.

### Stress-Stiffening Properties of Coiled Coil-Crosslinked PIC Networks

**Figure 3:**
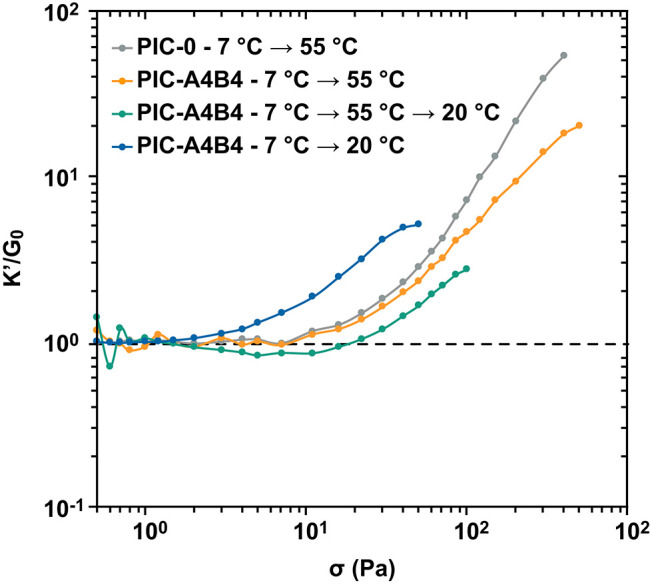
Stress-stiffening of PIC hydrogels. The PIC-A4B4 and PIC-0 samples (pre-treated with the respective temperature protocol) were subjected to a defined pre-stress σ and the normalized differential modulus *K*′/*G*_0_ was determined. The measurement was performed in triplicate. The additional data sets are shown in Supplementary Figure 4 and Supplementary Tables 3–6. Lines are drawn to guide the eye.

Subjecting the PIC-A4B4 sample to different temperature protocols has shown that the resulting network topology is different when the sample is maintained at temperatures below or above the LCST. In addition, a kinetically trapped bundle structure exists after the sample has been subjected to a heating-cooling cycle. PIC hydrogels are known to display stress-stiffening, which directly results from the presence of bundled, semi-flexible fiber structures. To gain insight into the effect of different network topologies on these non-linear viscoelastic properties, we determined the stress-stiffening response with rheology. We used an established pre-stress protocol where a small amplitude oscillatory stress is applied to the sample in the presence of a constant pre-stress. Performing a series of such measurements, the pre-stress is gradually increased. We measured the oscillatory strain response at a specific frequency (1 s^−1^). This method is gentle to the sample and recommended for studying transient material responses in the non-linear regime (Broedersz et al., 2010; Kouwer et al., 2013). It provides the normalized differential modulus (*K*′/*G*_0_; see Materials and Methods for details) as a function of the applied pre-stress (Figure 3). Two characteristic parameters can be extracted from this data to describe the stress-stiffening response. These are the slope or stiffening index *m*, which describes the intensity of the material response to the applied stress, and the critical stress σ_c_. The latter provides the stress value where the non-linear response sets in and thus describes the mechanosensitivity of the material.

Before starting the pre-stress protocol, the PIC-A4B4 sample was subjected to 3 different temperature protocols to obtain the above-mentioned network topologies: protocol 1 (7°C → 55°C) yields hydrophobically bundled networks stabilized by additional CC crosslinks; protocol 2 (7°C → 55°C → 20°C) results in kinetically trapped bundles that lack hydrophobic interactions; protocol 3 (7°C → 20°C) yields the equilibrium structure solely formed via CC-crosslinking. In addition, a PIC-0 sample subjected to protocol 1 was used as a control to determine the stress-stiffening response in the absence of CC crosslinks.

The PIC-A4B4 and PIC-0 samples measured in the presence of hydrophobically stabilized bundles (55°C) show a highly similar stress-stiffening response (Figure 3 and Supplementary Figure 4). The only difference is a reduced slope/stiffening index (Table 1), which becomes more and more apparent with increasing pre-stress. A similar reduction in slope was observed for PIC networks crosslinked with virus capsids (Schoenmakers et al., 2018b). We conclude that the difference in stiffening index for PIC-A4B4 and PIC-0 originates from the rupture of CC crosslinks, while the overall stress-stiffening response of the hydrogel is determined by the hydrophobically bundled PIC network.

**Table 1:** Summary of parameters describing the properties of PIC-A4B4 and PIC-0 hydrogels.

**Sample**	**PIC-0**	**PIC-A4B4**	**PIC-A4B4**	**PIC-A4B4**
Preparation	Protocol 1: 7°C → 55°C 55°C constant	Protocol 1: 7°C → 55°C 55°C constant	Protocol 2: 7°C → 55°C 55°C constant 55°C → 20°C 20°C constant	Protocol 3: 7°C → 20°C 20°C constant
Crosslinks	Bundling	CC + bundling	CC	CC
*G*_0_ (Pa)	242 ± 18	265 ± 28	711 ± 182	69 ± 4
Stiffening index *m*	1.42 ± 0.03	1.01 ± 0.04	0.71 ± 0.05	0.68 ± 0.08
Critical stress σ_c_ (Pa)	24.0 ± 1.3	17.9 ± 1.7	44.6 ± 11.6	4.1 ± 0.6
Critical strain γ_c_ (%)	10	6.8	6.3	5.9

*The values represent the mean of 3 independent experiments ± the standard error of the mean (SEM)*.

The PIC-A4B4 samples subjected to protocols 2 or 3 show a stress-stiffening response in the absence of hydrophobically stabilized bundles; however, with a lower stiffening index (Figure 3, Supplementary Figure 4, Table 1, and Supplementary Tables 7–9). This confirms that these samples contain semi-flexible structures, but to a smaller extent. While the stiffening indices for the two samples are highly similar, a clear difference is observed for σ_c_ (4.1 Pa for the equilibrated sample and 44.6 Pa for the sample containing kinetically trapped bundles). This is a direct result of the different temperature histories of these samples. The sample containing kinetically trapped bundles displays a plateau modulus *G*_0_ of 711 Pa at the start of the pre-stress protocol. In contrast, *G*_0_ is only 69 Pa for the sample equilibrated at 20°C. The observed difference in σ_c_ can be directly related to the critical strain γ_c_ using Hooke's Law: *G*_0_ = σ_c_/γ_c_. This relationship was used before to explain the stress-stiffening behavior of different PIC networks (Jaspers et al., 2014). Knowing *G*_0_ and σ_c_, we can thus calculate γ_c_, which is ~6% for both samples. This clearly shows that the non-linear response of these networks, which are stabilized by CC crosslinks only, sets in at the same applied strain and is determined by the CC-crosslinked network structure.

### Material Failure of Coiled Coil-Crosslinked PIC Hydrogels

**Figure 4:**
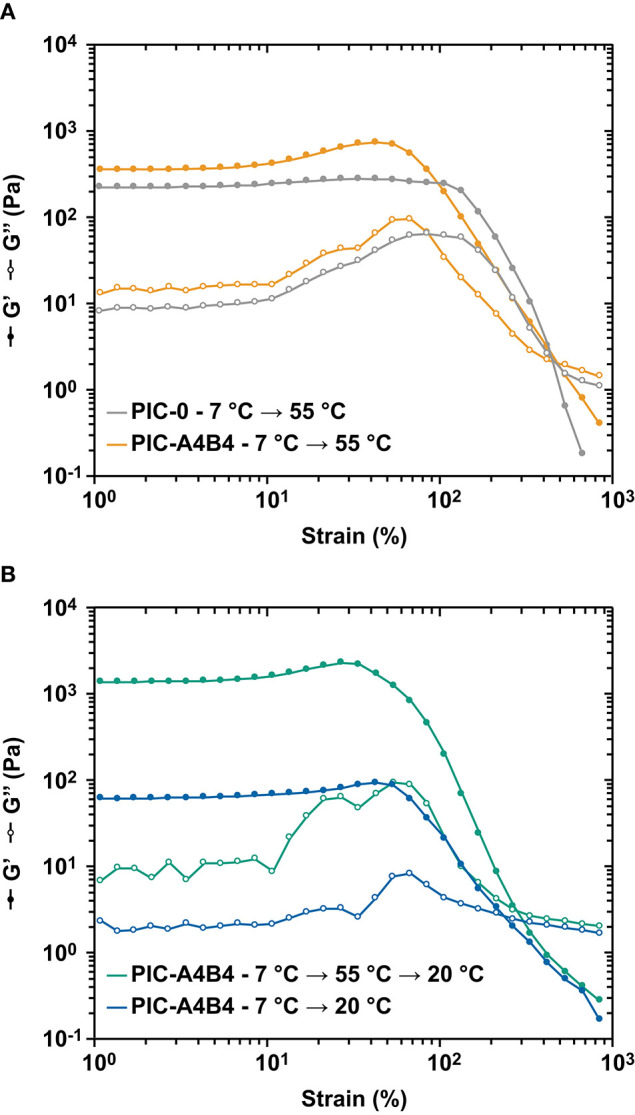
Amplitude sweeps of PIC. **(A)** Comparison of PIC-A4B4 and PIC-0 measured above the LCST at 55°C. **(B)** Comparison of the PIC-A4B4 sample equilibrated at 20°C and the PIC-A4B4 sample with trapped bundles, measured at 20°C. Each amplitude sweep was performed at a constant frequency of 1.6 s^−1^ while the strain amplitude γ was varied from 1 to 1,000%. Each measurement was performed in triplicate. The additional data sets are shown in Supplementary Figure 5. Lines are drawn to guide the eye.

The non-linear rheology experiments described above suggest that material failure is determined by the interactions that stabilize the network. If hydrophobically stabilized bundles are present, they dominate the material response and the respective materials fail at a higher stress than samples that contain CC crosslinks only (Figure 3). This suggests that the CC crosslinks break before the hydrophobically stabilized bundles disintegrate, as indicated by the different stiffening index for PIC-0 and PIC-A4B4 networks measured above the LCST. To confirm this result, we performed amplitude sweeps with the goal of determining the linear viscoelastic range (Figure 4 and Supplementary Figure 5). Again, we observed that the PIC-A4B4 sample measured above the LCST possessed similar properties as the PIC-0 sample (Figure 4A). Also in this experiment, the PIC-A4B4 containing hydrophobically stabilized bundles (55°C) tolerated larger deformation than the PIC-A4B4 hydrogels only stabilized by CC crosslinks (20°C) (Figure 4B). It is interesting to note that the equilibrated PIC-A4B4 sample and the kinetically trapped sample fail in a highly similar strain range, even though their *G*_0_ differs ~10-fold. This may suggest that the force propagates through the network via individual polymer chains and that the spatial organization of elastically active crosslinks is similar in both hydrogels. Lacking structural information about these networks, however, it is impossible to derive any further conclusions about their failure mechanism.

### Stress Relaxation in Coiled Coil-Crosslinked PIC and PEG Hydrogels

**Figure 5:**
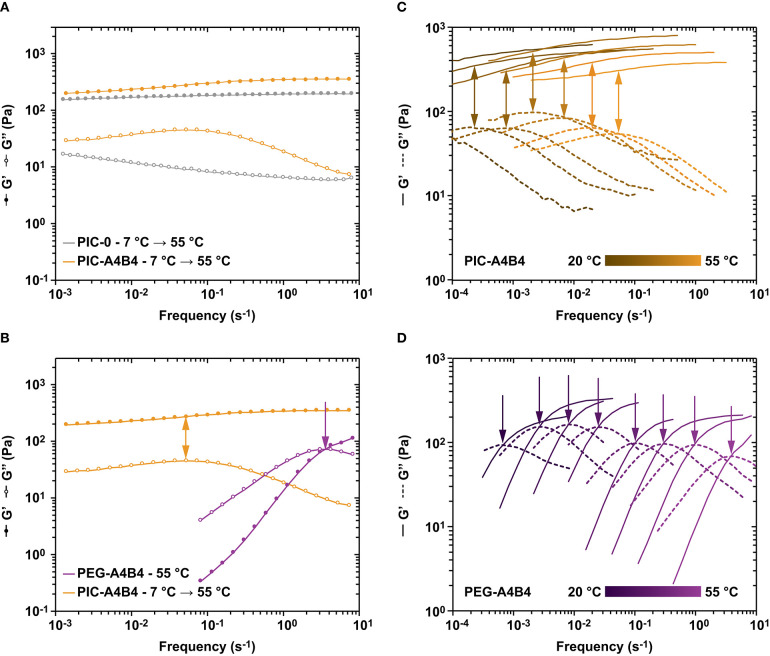
Frequency sweeps of PIC and PEG hydrogels. **(A)** Comparison of PIC-A4B4 and PIC-0 subjected to temperature protocol 1. Lines are drawn to guide the eye. **(B)** Comparison of PIC-A4B4 (temperature protocol 1) and PEG-A4B4 (measured at 55°C). Lines are drawn to guide the eye. **(C)** Comparison of the viscoelastic properties of PIC-A4B4 at different temperatures, ranging from 20 to 55°C. **(D)** Comparison of the viscoelastic properties of PEG-A4B4 at different temperatures, ranging from 20 to 55°C. For the PIC hydrogels, each frequency sweep was performed at a strain amplitude of 1%, while it was set to 10% for the PEG hydrogels. These strain amplitudes are in the linear viscoelastic range of each respective hydrogel. The frequency was varied from 0.0001 to 10 s^−1^. Additional data sets are shown in Supplementary Figure 6.

For the following analysis of stress relaxation, we thus primarily focus on measurements above the LCST (55°C), where the structure of the PIC-A4B4 network is well-defined and contains hydrophobically stabilized bundles as well as CC crosslinks. To obtain information about the importance of network topology for stress relaxation, we compare the PIC-A4B4 hydrogel to terminally crosslinked starPEG, which serves as a well-characterized reference network. The frequency sweeps were performed at 55°C (Figure 5 and Supplementary Figures 6, 8). For the PIC-A4B4 and PIC-0 hydrogels, all frequency sweeps were performed at a strain amplitude of 1%, while a strain amplitude of 10% was used for the PEG-A4B4 hydrogels. Both strain amplitudes lie in the linear viscoelastic range as determined from amplitude sweeps (Supplementary Figures 5, 8).

When comparing the viscoelastic properties of PIC-A4B4 with PIC-0, two key differences are observed. For PIC-A4B4, *G*′ is increased at high frequencies when compared to low frequencies. In contrast, *G*′ is almost constant for PIC-0 over the entire frequency range tested (Figure 5A and Supplementary Figure 7). For PIC-A4B4, a local maximum in the loss modulus *G*″ is further observed at a frequency of ~0.1 s^−1^, while this maximum is absent in the PIC-0 sample. Interestingly, the position of the local maximum coincides with the mentioned increase in *G*′ (Figure 5A).

A comparison with PEG-A4B4 provides more detailed insights into the origin of these features. We have shown earlier that the viscoelastic properties of CC-crosslinked starPEG networks are well-described with the Maxwell model (Tunn et al., 2018, 2019). These networks show a crossover between *G*′ and *G*″ at a characteristic frequency *f*_max_. This frequency correlates with the kinetic properties of the crosslinks (Grindy et al., 2015; Tunn et al., 2018). At frequencies smaller *f*_max_, *G*″ > *G*′ and the material behaves like a viscous liquid. At frequencies larger *f*_max_, *G*″ < *G*′ and the material behaves like an elastic solid (Figure 5B). *f*_max_ is directly related to the relaxation time τ of the material via τ = 1/*f*_max_. In other words, the CC crosslinks only contribute to the stability of the network at high frequencies where their kinetics is slower than the timescale of the applied oscillation. We thus assign the local maximum observed for PIC-A4B4 to the dissipative contribution of the CC crosslinks even though no crossover between *G*′ and *G*″ is observed. The macroscopic relaxation behavior is still largely determined by the properties of the PIC network even though the presence of CC crosslinks increases the stiffness of the material by 60% at the highest frequency tested (Supplementary Table 10).

Further proof for this interpretation was obtained from frequency sweeps performed over a range of temperatures from 55 to 20°C (Figures 5C,D). For both PIC-A4B4 and PEG-A4B4, a shift of the *G*″ maximum to lower frequencies is observed. This is expected as the crosslink kinetics becomes slower with decreasing temperature. It should be noted that a detailed interpretation of this data is difficult for PIC-A4B4, however, as the kinetically trapped network rearranges during the measurement. Furthermore, the LCST transition is crossed so that contributions from hydrophobic PIC bundling are hidden just as temperature-dependent changes in the *L*_p_ of individual PIC polymers and bundles. Despite the contribution of these unquantified additional factors, the shift of the *G*″ maximum with temperature is similar for PIC-A4B4 and PEG-A4B4 samples.

The most striking result of the PIC-A4B4 and PEG-A4B4 comparison is the clear difference in relaxation times between these networks over the entire temperature range tested. At a temperature of 55°C, the relaxation time differs by approximately two orders of magnitude, even though the crosslink used is exactly the same. This suggests that crosslink kinetics is not the only parameter that determines the relaxation time of a material. In the PEG-A4B4 network, two PEG arms are terminally connected via exactly one crosslink. In the absence of entanglements, crosslink dissociation therefore immediately relaxes an elastically active chain. In a well-crosslinked starPEG network, stress relaxation should thus indeed be mostly determined by the dissociation rate of the crosslink [*k*_off_ = 3.2 · 10^−4^ s^−1^ at 25°C (Goktas et al., 2018)]. Our results show *f*_max_ of ~2.5 · 10^−3^ s^−1^ at 25°C, which is one order of magnitude higher than *k*_off_. This difference is explained by the presence of network defects, such as loops and superchains, which are known to speed up network relaxation (Annable et al., 1993; Rossow et al., 2014; Ciarella et al., 2018).

The network topology of PIC-A4B4 at 55°C is very different. In hydrophobically stabilized bundles, the CCs form multiple crosslinks within the bundles. Considering a mean polymer length of 412 nm and an average CC spacing of ~14 nm, one polymer is connected within the bundle via ~30 crosslinks. In addition, each bundle is estimated to consist of 7–9 polymers so that the next crosslink is on average found within <4 nm along the bundle. Further considering the high *L*_p_ of the polymers, re-association of dissociated CCs is thus easily possible. It is, therefore, extremely unlikely that the dissociation of one CC crosslink allows relaxation of a polymer chain or even the entire bundle. Stress relaxation instead requires the dissociation of several crosslinks simultaneously. Multivalent binding of CC crosslinks, determined by the topology of the bundled PIC network, is thus the most likely explanation for the observed increase in the relaxation time.

Based on this knowledge, we now compare the relaxation time of PIC-A4B4 networks below the LCST where hydrophobically stabilized bundles are absent (Supplementary Figures 6D,E). Also, for these networks the relaxation time is significantly lower than for PEG-A4B4 (Supplementary Figures 6C, 8B). For the PIC-A4B4 hydrogels measured at 20°C, no local maximum in *G*″ is visible in the accessible frequency range and the maximum is most likely located at much lower frequencies. This suggests that the relaxation of elastically active chains is also hindered by multivalent CC interactions at 20°C, confirming our earlier interpretation that PIC-A4B4 networks also contain bundle-like structures below the LCST. In fact, no significant difference in the frequency dependence is observed for the network equilibrated at 20°C and the network containing kinetically trapped bundles, suggesting that elastically active chains relax in a similar fashion. Even though we are not able to quantitatively compare the relaxation times, our combined results provide sufficient evidence to conclude that the combination of network topology and crosslink kinetics determines the relaxation behavior of elastically active chains in dynamically crosslinked hydrogel networks.

## Conclusions

Introducing CC crosslinks into PIC and starPEG networks has allowed us to directly compare stress relaxation in dynamically crosslinked hydrogels of different network topology. The CC-crosslinked and fiber-like PIC network retains its stress-stiffening properties and displays a relaxation time approximately two orders of magnitude longer than the starPEG reference network. This clearly shows that stress relaxation is determined by a combination of crosslink kinetics and network topology. In the PIC hydrogel, each polymer is connected to the network via multiple CC crosslinks. Stress relaxation of elastically active chains thus requires the simultaneous dissociation of several CCs. In contrast, each elastically active chain is connected by exactly one crosslink in the starPEG network. Controlling the number of crosslinks per chain thus appears to be a possible new design principle for tuning the viscoelastic properties of synthetic polymeric materials. Especially when combined with semi-flexible polymers, adjusting the distance between crosslinks to the persistence length may facilitate the formation of fiber bundles and introduce stress-stiffening behavior into such materials. Hierarchical assembly, stress relaxation and non-linear stress-strain responses are thus tightly connected and can only be engineered in combination. Nature has elegantly utilized this concept in the cytoskeleton and the ECM. These structures show slow macroscopic stress relaxation, but contain fine-tuned dynamic crosslinks that facilitate local network reorganization. This specific interplay between macroscopic and microscopic properties allows cell motility and spreading while maintaining the overall shape of tissues. The CC-crosslinked PIC network serves as an excellent model system to tune crosslink density, kinetics, and thermodynamics independently. This may ultimately provide a stress-stiffening network for cell culture experiments where macroscopic stress relaxation and local crosslink dynamics are decoupled.

## Data Availability Statement

The datasets generated for this study can be found in Edmond — the Open Access Data Repository of the Max Planck Society (https://dx.doi.org/10.17617/3.3z).

## Author Contributions

EG, AL, RH, and KB: conceptualization and validation. EG, IT, DV, and AL: investigation. KB: resources and project administration. EG, IT, and KB: data curation and visualization. EG: writing—original draft. AL, RH, and KB: writing—review and editing. RH, AL, and KB: supervision. AL and KB: funding acquisition. All authors have given approval to the final version of the manuscript.

## Conflict of Interest

The authors declare that the research was conducted in the absence of any commercial or financial relationships that could be construed as a potential conflict of interest.
