# Supplementary material for: Influence of Network Topology on the Viscoelastic Properties of Dynamically Crosslinked Hydrogels
Source: Front Chem. 2020 Jun 30;8:536. doi: 10.3389/fchem.2020.00536 (PMC7349520; doi:10.3389/fchem.2020.00536)
Supplement: Supplementary file 1 [file Data_Sheet_1.pdf]

Supplementary Material

Influence of network topology on the viscoelastic properties of dynamically crosslinked hydrogels

Emilia M. Grad[1,2], Isabell Tunn[1], Dion Voerman[3,4], Alberto S. de Léon[1†*], Roel Hammink[3,4*], Kerstin G. Blank[1,2*]

[1]Mechano(bio)chemistry, Max Planck Institute of Colloids and Interfaces, Potsdam, Germany

[2]Department of Molecular Materials, Institute for Molecules and Materials, Radboud University, Nijmegen, The Netherlands

[3]Department of Tumor Immunology, Radboud Institute for Molecular Life Sciences, Radboud University Medical Center, Nijmegen, The Netherlands

[4]Division of Immunotherapy, Oncode Institute, Radboud University Medical Center, Nijmegen, The Netherlands

[†]present address: Department of Materials Science, University of Cádiz, Cádiz, Spain

***Correspondence:**
Alberto S. de Léon: alberto.sanzdeleon@uca.es
Roel Hammink: roel.hammink@radboudUMC.nl
Kerstin G. Blank: kerstin.blank@mpikg.mpg.de

Table of Contents

1. MALDI-TOF analysis of the DBCO-functionalized CC-A4B4 ..... S3
2. Formation of the PIC-0 and PIC-A4B4 hydrogels at 20 °C..... S4
3. Repeated heating-cooling cycles of the PIC-A4B4 hydrogel S5
4. Detection of hydrophobic PIC bundling with Nile Red..... S6
5. Non-linear rheology of the PIC hydrogels..... S7
6. Amplitude sweeps of the PIC and PEG hydrogels..... S14
7. Frequency sweeps of the PIC and PEG hydrogels..... S15
8. Comparison of measurement geometries for PEG hydrogels S18

1. MALDI-TOF analysis of the DBCO-functionalized CC-A4B4

The preformed CC-A4B4 (1 mM) was incubated with 2 mM DBCO-EG_4-maleimide (Cys:maleimide = 1:1), desalted and analyzed with MALDI-TOF mass spectrometry (α-cyano-4-hydroxycinnamic acid matrix; linear-positive mode). The main peaks represent the individual DBCO-functionalized peptides A4 and B4. A small amount (<10%) of unreacted peptides is also present (Figure S1). Considering the high yield of the coupling reaction, purification of the functionalized CC was omitted.

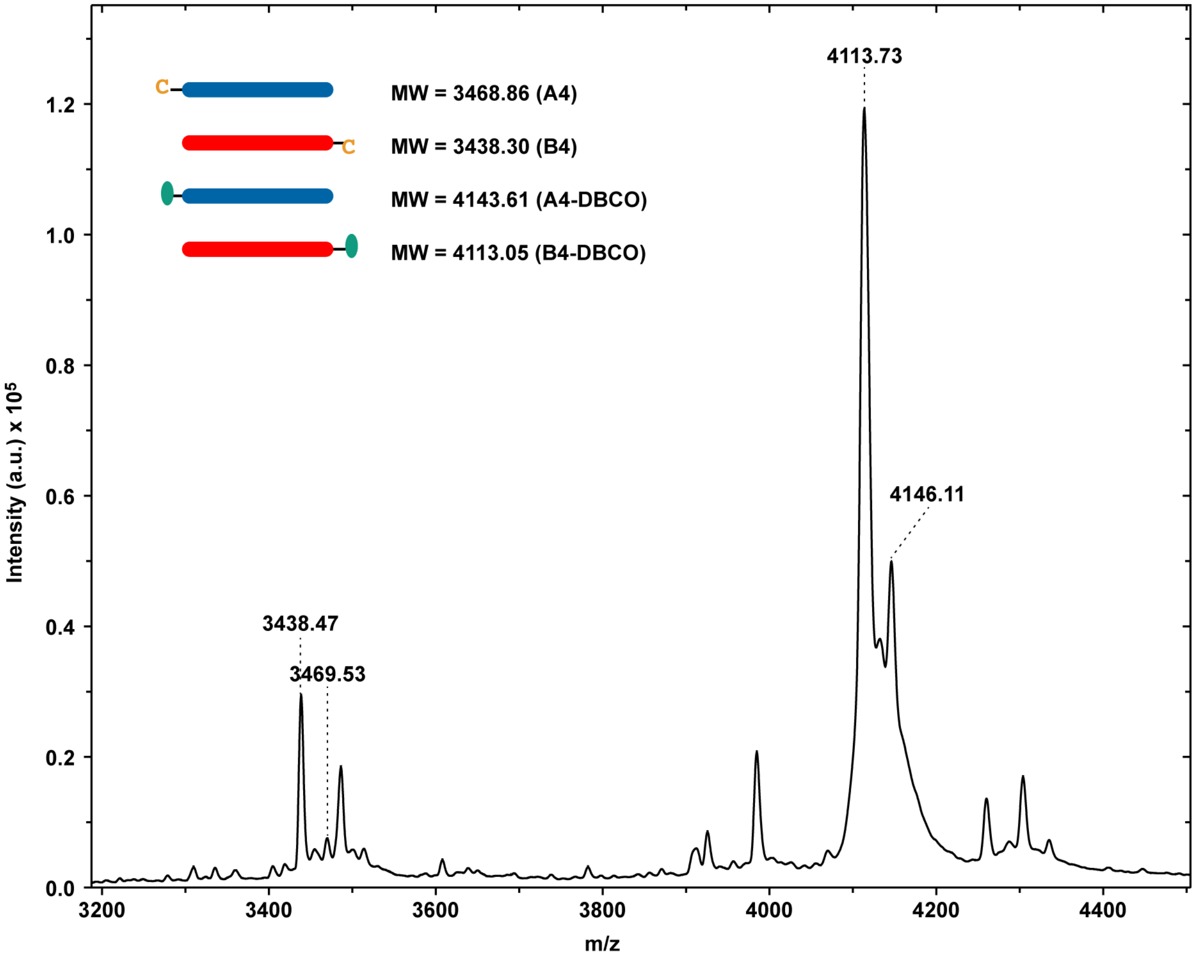

Supplementary figure 1. MALDI-TOF analysis of CC-A4B4 after reaction with DBCO-EG_4-maleimide (Cys:maleimide = 1:1).

2. Formation of the PIC-0 and PIC-A4B4 hydrogels at 20 °C

To prove that the addition of CC-A4B4 facilitates PIC crosslinking in the absence of hydrophobic bundling (i.e. below the LCST), the time evolution of the storage modulus *G*' was followed and compared to the pure PIC sample (PIC-0). Both samples were prepared as described in the main text and subjected to temperature protocol 3, while measuring *G*'. It is clearly evident that *G*' increased in the PIC-A4B4 sample upon increasing the temperature from 7 °C to 20 °C and reached a plateau after approximately 1-2 h (Figure S2) when the sample was maintained at 20 °C. In contrast, no increase in *G*' was observed in the PIC-0 sample tested under the same conditions.

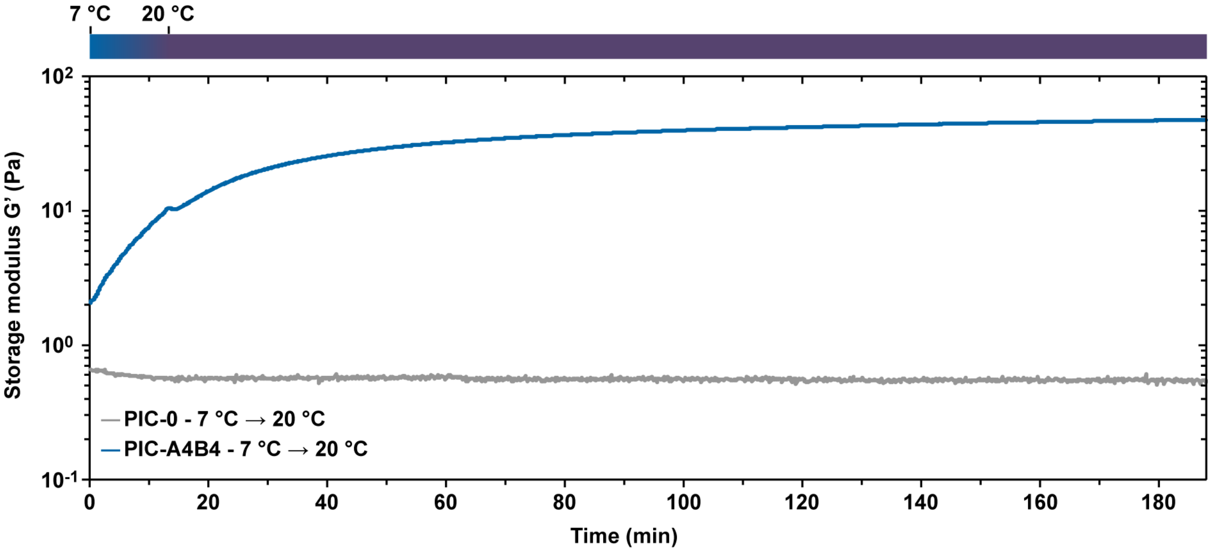

Supplementary figure 2. Time evolution of the storage modulus *G*' for PIC-A4B4 and PIC-0 when the samples are subjected to temperature protocol 3 (incubation below the LCST). The storage modulus was measured at a strain amplitude of 1 % (linear viscoelastic range) and a frequency of 1.6 s^{-1}.

3. Repeated heating-cooling cycles of the PIC-A4B4 hydrogel

To test the contribution of CC-A4B4 to the formation, stabilization and disassembly of bundled PIC-A4B4 networks, the sample was first heated to 55 °C and incubated at this temperature for 90 min to induce hydrophobic bundling and to facilitate crosslinking of the PIC bundles with CC-A4B4. The sample was then cooled to 20 °C and kept at this temperature for 90 min. This corresponds to protocol 2 with an extended incubation time at 20 °C. To further test for the reversibility of network assembly and disassembly, this temperature cycle was repeated 4x (Figure S3).

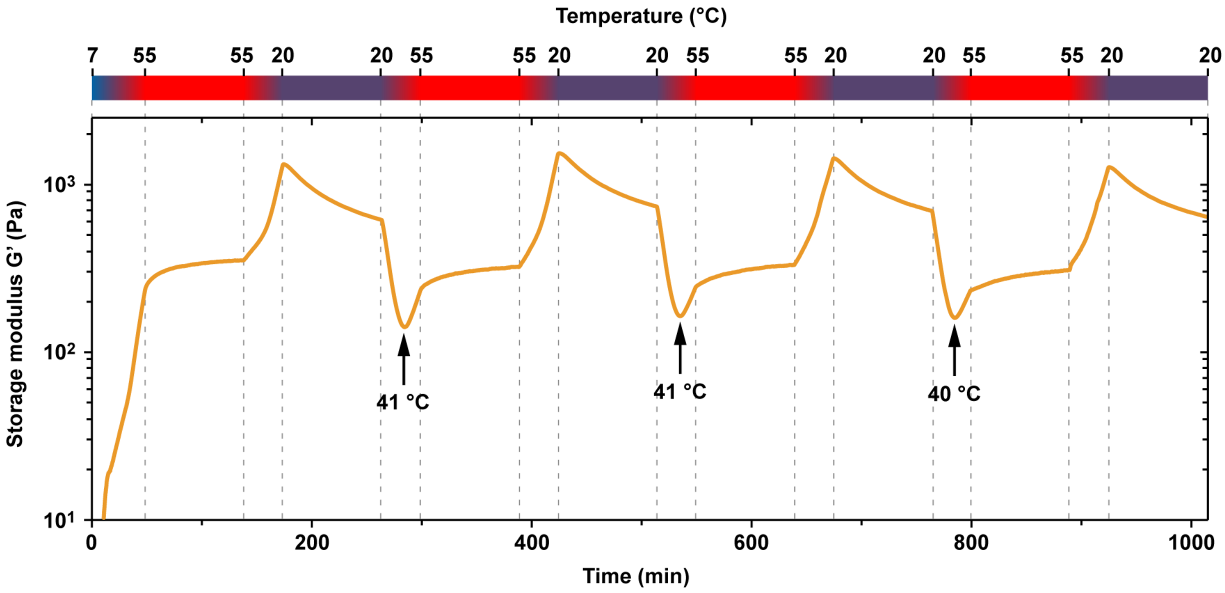

Supplementary figure 3. Repeated heating and cooling cycles for PIC-A4B4. The sample was heated from 7 °C to 55 °C with a rate of 1 °C min^{-1} and kept at 55 °C for 90 min. It was then cooled from 55 °C to 20 °C at a rate of 1 °C min^{-1} and kept at 20 °C for 90 min (extended protocol 2). The time evolution of the storage modulus *G*' was followed during the entire temperature protocol. *G*' was measured in an oscillatory experiment with a strain amplitude in the linear viscoelastic range (1 %) and a frequency of 1.6 s^{-1}.

In all cycles, *G*' initially increased upon cooling. It then decreased gradually when the sample was held constant at 20 °C. This decrease continued with a steeper slope when the sample was again heated to 55 °C. Once the LCST was reached, *G*' increased to approximately the same value in all temperature cycles. We attribute this behavior to the presence of the CC crosslinks. CC dissociation and PIC debundling are both reversible; however, at very different time scales. While the LCST transition is expected to be fast, CC-A4B4 has a half-life ($\ln2/k_{off}$) of approximately 35 min at 25 °C ($k_{off} = 3.2 \cdot 10^{-4}$ s^{-1}). The initial increase in *G*' upon cooling most likely originates from a higher thermodynamic stability of the CC crosslinks at lower temperatures, which increases the total number of CC crosslinks in the network. It should be noted that the time interval where the temperature is held constant at 20 °C could possibly be used to obtain the 'rate of debundling'. We have not attempted to fit these data, as also the PIC persistence length is temperature-dependent. The data in Figure S3 thus provide only qualitative information about CC dissociation and debundling.

Remarkably, no difference was observed between the first and the following heating-cooling cycles. This suggests that the final network properties at 55 °C are independent of the type of crosslinking reaction. In the first cycle, the reaction between azide and DBCO causes crosslinking, while CC-A4B4 association is responsible for crosslink formation in subsequent temperature cycles.

4. Detection of hydrophobic PIC bundling with Nile Red

To prove that no hydrophobically stabilized bundles are present when the CC crosslinked samples were cooled below the LCST, the dye Nile Red was incubated with the samples before, during and after crosslinking the PIC bundles with the CC. Nile Red fluorescence is known to increase when the dye experiences a hydrophobic environment.

Supplementary table 1. Nile Red fluorescence at different temperatures below and above the LCST. Values in brackets represent the relative increase in Nile Red fluorescence, compared to the initial fluorescence intensity at 30 °C.

**Temperature (°C)**	**Nile Red fluorescence @ 655 nm (a. u.)**	
	PIC-0	PIC-A4B4
30	652 (100 %)	851 (100 %)
35	693	1004
40	826	1180
45	1169	1445
50	1670	1877
55	6098 (940 %)	5262 (620 %)
40	901	2086
30	612 (94 %)	1170 (140 %)

Nile Red fluorescence increased when the temperature was raised from 30 °C to 55 °C and decreased again when the sample was cooled down to 30 °C, both for PIC-A4B4 and the PIC-0 control sample without CC crosslinks (Table S1). This clearly shows that the hydrophobicity of the polymer decreases when cooling the sample below the LCST in both cases. When comparing the fluorescence intensity of PIC-A4B4 with PIC-0 at 55 °C it is observed that the relative Nile Red intensity is lower for PIC-A4B4, suggesting that the CC crosslinks slightly interfere with hydrophobic bundle formation and stabilization. After cooling the bundled samples to 30 °C, the comparison shows a minimally higher relative fluorescence for PIC-A4B4. This may suggest that crosslinking keeps hydrophobic polymers in close proximity thereby slowing down the LCST.

5. Non-linear rheology of the PIC hydrogels

To detect and quantify stress-stiffening in the PIC hydrogels, non-linear rheology was performed as described in the Materials and Methods section and in Table S2, which summarizes all applied pre-stress values as well as each respective oscillatory stress. From these pre-stress experiments, the differential modulus $K' = \delta\sigma/\delta\gamma$ was determined for 3 independent samples (Tables S3-S6).

Supplementary table 2. Pre-stress protocol for non-linear rheology. The amplitude of the superposed oscillatory stress was ≤10 % of each respective pre-stress value.

Pre-stress σ (Pa)	Oscillatory stress δσ (Pa)	Frequency range (s^{-1})
0.5	0.05	0.1 – 10
0.6	0.05	0.1 – 10
0.7	0.05	0.1 – 10
0.8	0.05	0.1 – 10
1	0.10	0.1 – 10
1.2	0.10	0.1 – 10
1.5	0.10	0.1 – 10
2	0.10	0.1 – 10
3	0.10	0.1 – 10
4	0.10	0.1 – 10
5	0.10	0.1 – 10
7	0.10	0.1 – 10
11	0.10	0.1 – 10
16	0.10	0.1 – 10
22	1.00	0.1 – 10
30	1.00	0.1 – 10
40	1.00	0.1 – 10
50	1.00	0.1 – 10
60	1.00	0.1 – 10
70	1.00	0.1 – 10
85	1.00	0.1 – 10
100	1.00	0.1 – 10
120	1.00	0.1 – 10
150	1.00	0.1 – 10
200	2.00	0.1 – 10
300	3.00	0.1 – 10
400	4.00	0.1 – 10
500	5.00	0.1 – 10
600	6.00	0.1 – 10

Supplementary table 3. Differential moduli ($K' = \delta\sigma/\delta\gamma$) calculated for the PIC-0 hydrogel (temperature protocol 1). The $\delta\gamma$ values were extracted from the raw data at a frequency of 1 s^{-1}. For $\delta\sigma$, the pre-set oscillatory stress values were used (see Table S2). Each measurement was performed in triplicate, using a freshly prepared hydrogel sample. Data points taken after material failure are indicated (-).

**Pre-stress σ (Pa)**	**Differential modulus *K'* (Pa)**		
	Sample 1	Sample 2	Sample 3
0.5	0.98	0.95	0.94
0.6	0.98	0.97	0.94
0.7	0.99	0.96	0.91
0.8	1.01	0.97	0.94
1	0.99	0.99	0.97
1.2	1.01	1.00	0.94
1.5	0.99	0.99	1.08
2	0.96	0.97	0.95
3	0.99	1.01	0.95
4	1.02	1.01	0.95
5	1.02	1.00	0.96
7	0.97	1.07	1.06
11	1.15	1.12	1.10
16	1.25	1.26	1.32
22	1.47	1.40	1.58
30	1.81	1.67	2.03
40	2.25	2.04	2.59
50	2.83	2.50	3.22
60	3.47	3.01	4.15
70	4.21	3.61	5.04
85	5.66	4.82	6.86
100	7.17	5.91	9.07
120	9.79	7.98	12.05
150	13.14	11.08	15.69
200	21.47	17.85	27.66
300	39.05	32.55	38.27
400	53.77	43.27	-
500	-	-	-
600	-	-	-

Supplementary table 4. Differential moduli ($K' = \delta\sigma/\delta\gamma$) calculated for the PIC-A4B4 hydrogel, prepared with temperature protocol 1. The $\delta\gamma$ values were extracted from the raw data at a frequency of 1 s^{-1}. For $\delta\sigma$, the pre-set oscillatory stress values were used (see Table S2). Each measurement was performed in triplicate, using a freshly prepared hydrogel sample. Data points taken after material failure are indicated (-).

**Pre-stress σ (Pa)**	**Differential modulus *K'* (Pa)**		
	Sample 1	Sample 2	Sample 3
0.5	1.16	0.88	0.95
0.6	0.99	0.92	0.97
0.7	0.96	0.59	0.93
0.8	0.88	0.96	0.97
1	0.93	1.01	1.00
1.2	1.10	0.94	0.97
1.5	0.97	1.04	1.01
2	0.92	0.99	1.23
3	1.04	0.82	1.07
4	0.95	0.98	0.86
5	0.99	1.12	1.09
7	0.94	1.21	1.09
11	1.10	1.11	1.13
16	1.18	1.26	1.53
22	1.34	1.50	1.75
30	1.61	1.81	2.20
40	1.96	2.23	2.90
50	2.32	2.73	3.44
60	2.83	3.29	4.46
70	3.17	3.81	5.21
85	4.06	4.49	6.40
100	4.57	5.46	7.55
120	5.40	6.61	9.11
150	7.10	8.16	11.94
200	9.29	11.01	16.65
300	13.86	14.82	25.10
400	17.97	17.29	34.00
500	20.12	-	38.88
600	-	-	-

Supplementary table 5. Differential moduli ($K' = \delta\sigma/\delta\gamma$) calculated for the PIC-A4B4 hydrogel, prepared with temperature protocol 2. The $\delta\gamma$ values were extracted from the raw data at a frequency of 1 s^{-1}. For $\delta\sigma$, the pre-set oscillatory stress values were used (see Table S2). Each measurement was performed in triplicate, using a freshly prepared hydrogel sample. Data points taken after material failure are indicated (-).

**Pre-stress σ (Pa)**	**Differential modulus *K*' (Pa)**		
	Sample 1	Sample 2	Sample 3
0.5	1.16	1.22	1.40
0.6	1.07	1.17	0.70
0.7	1.15	1.16	1.19
0.8	1.01	1.18	0.99
1	1.04	1.02	1.04
1.2	0.99	1.02	0.99
1.5	0.95	0.94	0.96
2	1.00	0.87	0.92
3	0.90	0.86	0.88
4	0.93	0.49	0.85
5	0.91	0.88	0.82
7	0.98	0.85	0.85
11	0.93	0.85	0.84
16	0.90	0.62	0.92
22	0.91	0.85	1.03
30	0.96	0.96	1.18
40	1.06	1.08	1.42
50	1.15	1.26	1.63
60	1.26	1.45	1.91
70	1.41	1.59	2.15
85	1.55	1.86	2.51
100	1.74	2.09	2.71
120	1.94	2.46	-
150	-	2.88	-
200	-	3.59	-
300	-	-	-
400	-	-	-
500	-	-	-
600	-	-	-

Supplementary table 6. Differential moduli ($K' = \delta\sigma/\delta\gamma$) calculated for the PIC-A4B4 hydrogel, prepared with temperature protocol 3. The $\delta\gamma$ values were extracted from the raw data at a frequency of 1 s^{-1}. For $\delta\sigma$, the pre-set oscillatory stress values were used (see Table S2). Each measurement was performed in triplicate, using a freshly prepared hydrogel sample. Data points taken after material failure are indicated (-).

**Pre-stress σ (Pa)**	**Differential modulus *K*' (Pa)**		
	Sample 1	Sample 2	Sample 3
0.5	0.99	0.96	0.97
0.6	0.98	0.97	0.95
0.7	0.97	0.97	0.96
0.8	0.98	0.97	0.96
1	0.99	0.98	0.97
1.2	0.99	0.98	0.99
1.5	1.01	1.01	1.02
2	1.04	1.03	1.03
3	1.11	1.13	1.10
4	1.18	1.20	1.39
5	1.29	1.31	1.30
7	1.48	1.49	1.48
11	1.84	1.99	1.96
16	2.44	2.47	2.56
22	3.15	-	3.28
30	4.11	-	4.34
40	4.83	-	5.46
50	5.06	-	6.47
60	-	-	6.87
70	-	-	-
85	-	-	-
100	-	-	-
120	-	-	-
150	-	-	-
200	-	-	-
300	-	-	-
400	-	-	-
500	-	-	-
600	-	-	-

In the following, the differential moduli K' were normalized to the plateau moduli G_0, which were obtained from G' in the linear viscoelastic range. The G_0 values for all three independent samples as well as their mean and standard error of the mean (SEM) are shown in Table S7. The normalized K'/G_0 values were then plotted against the applied pre-stress σ for all three samples (Figure S4).

Supplementary table 7. Plateau moduli G_0 for the different PIC hydrogels. The G_0 values were obtained by averaging over the measured storage moduli at pre-stress values of 1, 1.5 and 2 Pa. These pre-stress values are in the linear viscoelastic range for each of the hydrogel samples. Each measurement was performed in triplicate, using a freshly prepared hydrogel sample. The values for each individual sample as well as their mean and standard error of the mean (SEM) are shown.

**Sample**	**Plateau modulus G_0 (Pa)**			
	PIC-0	PIC-A4B4 (P1)	PIC-A4B4 (P2)	PIC-A4B4 (P3)
1	244	411	653	72
2	272	370	568	62
3	210	314	390	74
**Mean**	**242**	**365**	**537**	**69**
**SEM**	**18**	**28**	**77**	**4**

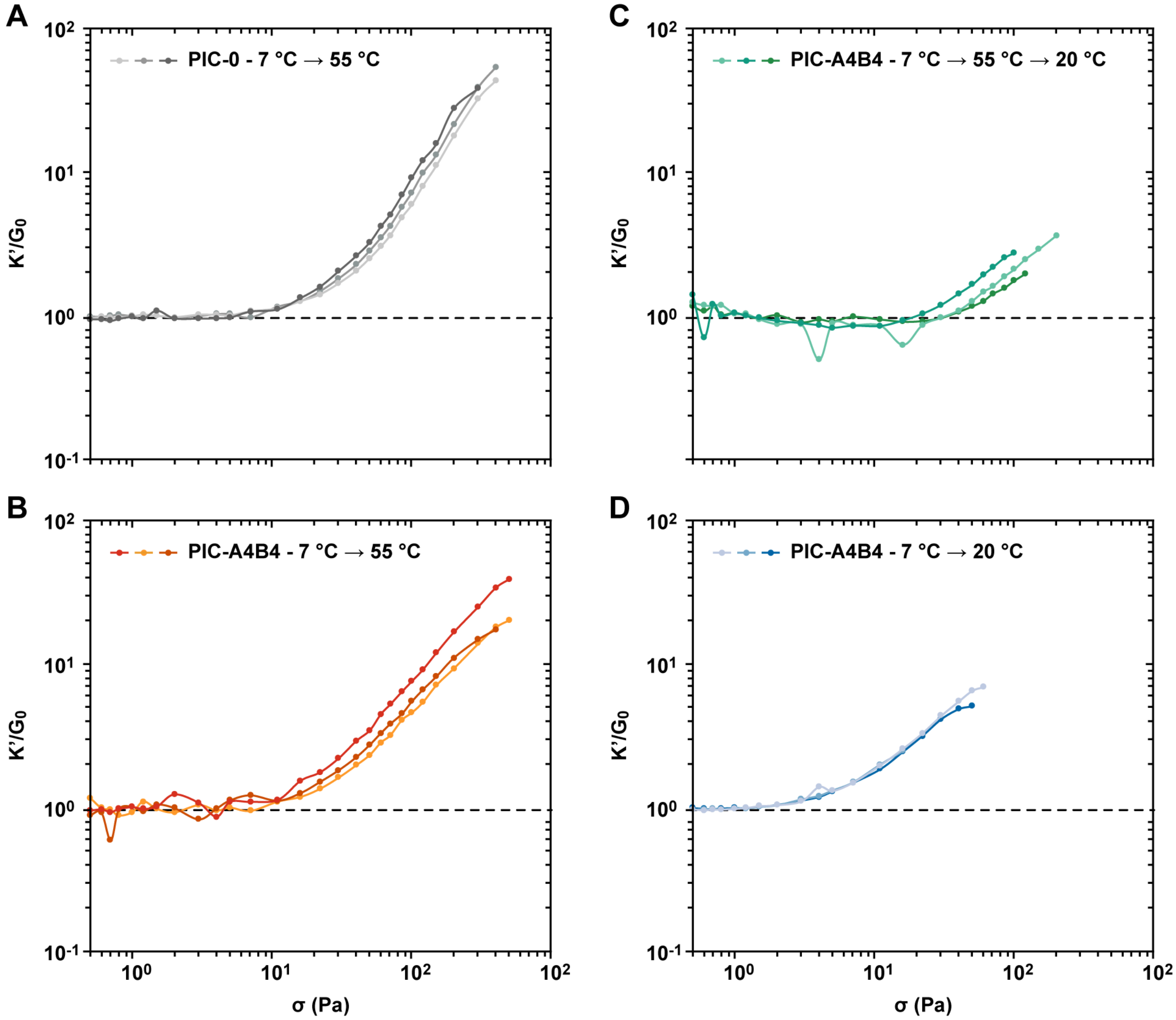

Supplementary figure 4. Non-linear rheology of the different PIC hydrogels. (A) PIC-0 subjected to temperature protocol 1. (B) PIC-A4B4 subjected to temperature protocol 1. (C) PIC-A4B4 subjected to temperature protocol 2. (D) PIC-A4B4 subjected to temperature protocol 3. Shown are three independent measurements, each performed with a freshly prepared hydrogel sample. Lines are drawn to guide the eye.

To characterize the non-linear response of each hydrogel, the stiffening index *m* (Table S8) as well as the critical stress σ_c (Table S9) were determined for all 3 independent samples of each hydrogel.

Supplementary table 8. Stiffening index *m* for the different PIC hydrogels. The values for *m* were obtained from the slope of the K'/G_0 vs. σ plots. The values for each individual sample as well as their mean and standard error of the mean (SEM) are shown.

**Sample**	**Stiffening index *m***			
	PIC-0	PIC-A4B4 (P1)	PIC-A4B4 (P2)	PIC-A4B4 (P3)
1	1.39	0.98	0.57	0.76
2	1.40	0.97	0.75	0.53
3	1.48	1.08	0.74	0.76
**Mean**	**1.42**	**1.01**	**0.69**	**0.68**
**SEM**	**0.03**	**0.04**	**0.07**	**0.08**

Supplementary table 9. Critical stress σ_c for the different PIC hydrogels. The values for each individual sample as well as their mean and standard error of the mean (SEM) are shown.

**Sample**	**Critical stress σ_c**			
	PIC-0	PIC-A4B4 (P1)	PIC-A4B4 (P2)	PIC-A4B4 (P3)
1	22.9	20.9	38.3	4.9
2	26.5	17.5	36.8	3.0
3	22.5	15.2	25.0	4.5
**Mean**	**24.0**	**17.9**	**33.4**	**4.1**
**SEM**	**1.3**	**1.7**	**4.2**	**0.6**

6. Amplitude sweeps of the PIC and PEG hydrogels

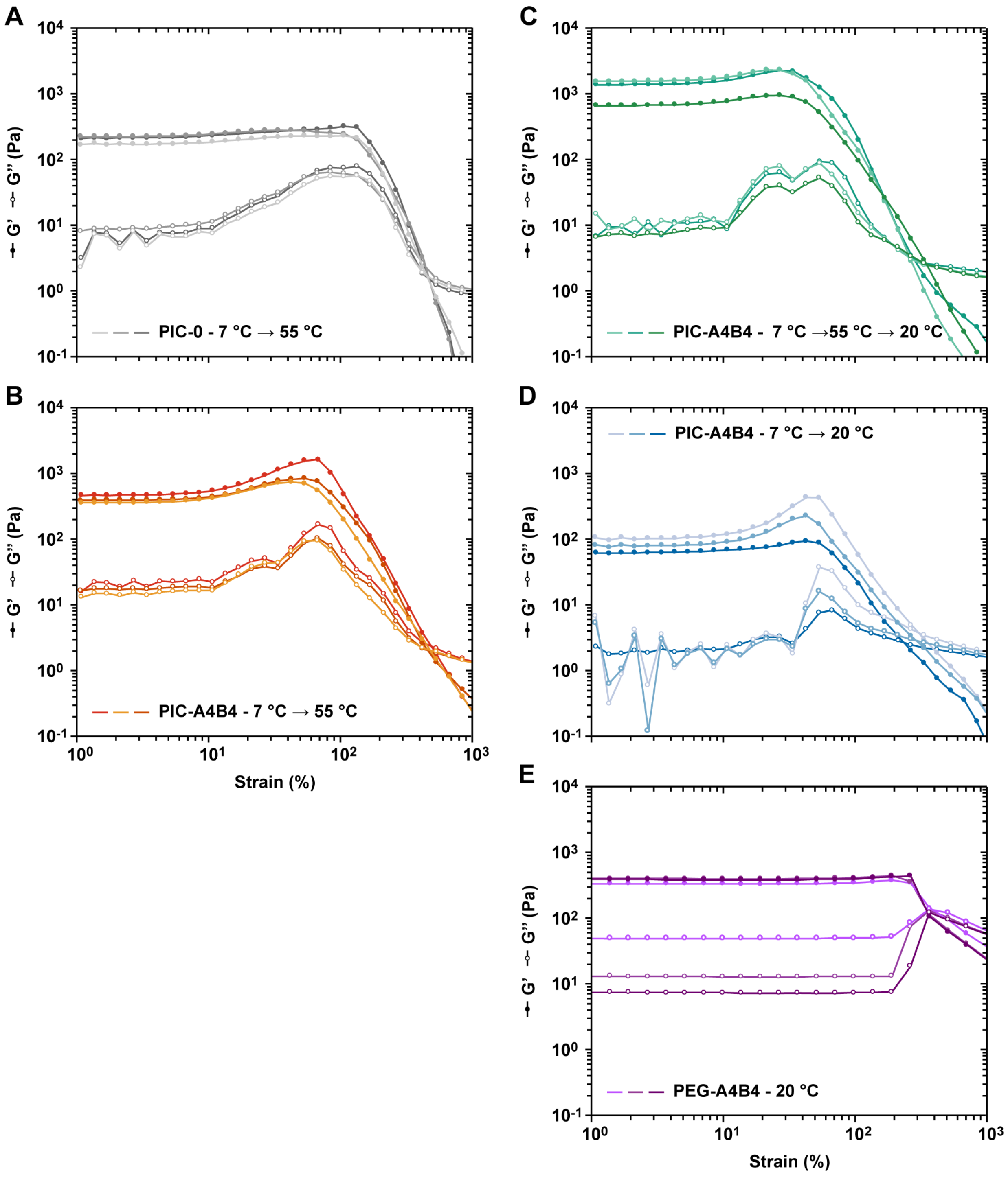

Supplementary figure 5. Amplitude sweeps of all hydrogels. (A) PIC-0 subjected to temperature protocol 1. (B) PIC-A4B4 subjected to temperature protocol 1. (C) PIC-A4B4 subjected to temperature protocol 2. (D) PIC-A4B4 subjected to temperature protocol 3. (E) PEG-A4B4 hydrogel measured at 20 °C. Each amplitude sweep was performed at a frequency of 1.6 s^{-1}. Shown are three independent measurements, each performed with a freshly prepared hydrogel sample. Lines are drawn to guide the eye.

7. Frequency sweeps of the PIC and PEG hydrogels

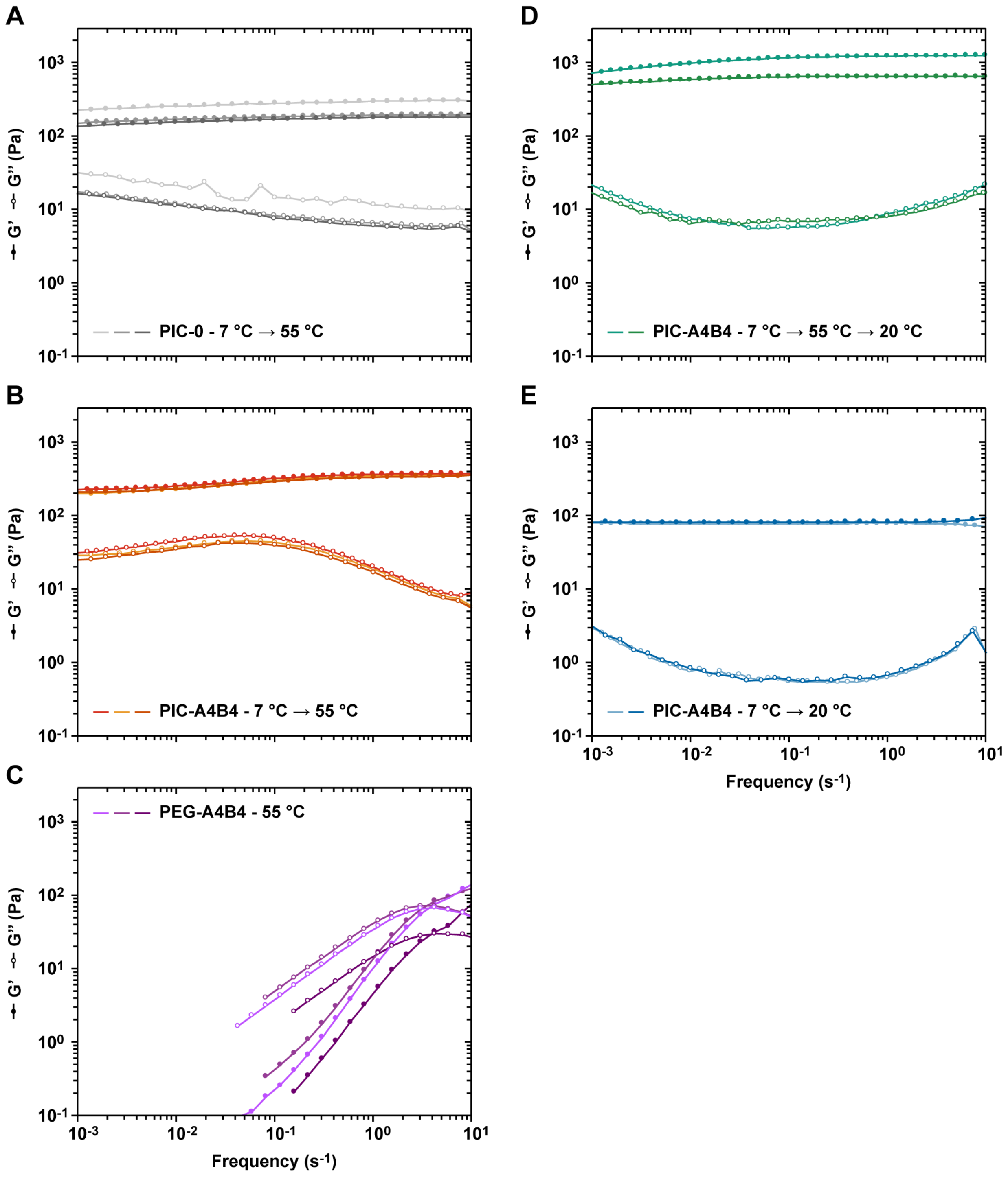

Supplementary figure 6. Frequency sweeps of all hydrogels. (A) PIC-0 subjected to temperature protocol 1. (B) PIC-A4B4 subjected to temperature protocol 1. (C) PEG-A4B4 hydrogel at 55 °C. (D) PIC-A4B4 subjected to temperature protocol 2 (n = 2). (E) PIC-A4B4 subjected to temperature protocol 3 (n = 2). For the PIC hydrogels, a strain amplitude of 1 % was used, while it was set to 10 % for the PEG-A4B4 hydrogel. Shown are three independent measurements, each performed with a freshly prepared hydrogel sample. Lines are drawn to guide the eye.

To visualize the contribution of the dynamic CC crosslinks to the hydrophobically bundled PIC network more clearly, the data for PIC-0 and PIC-A4B4 are compared in the same graph (Figure 5A and Figure S7A). A clear maximum is visible for the loss modulus *G*'', which is considered to be correlated to the relaxation time τ ($f_{max} = 1/\tau$). At f_{max}, also an increase in *G*' is observed (Figure S7). This is assigned to a stronger contribution of the CC crosslinks to network stabilization at frequencies above f_{max}.

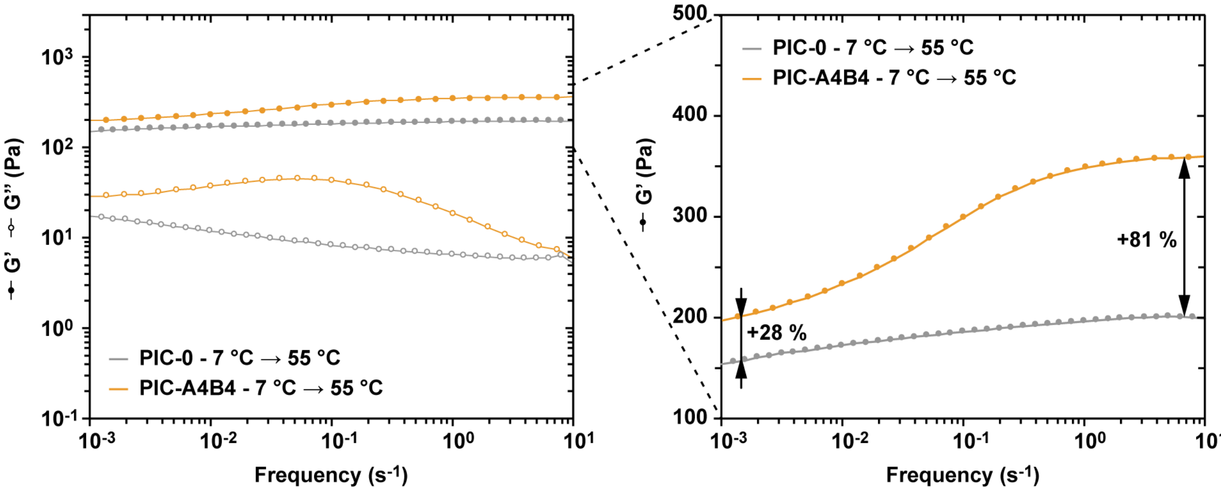

Supplementary figure 7. Comparison of CC crosslinked PIC-A4B4 with non-crosslinked PIC (PIC-0) at 55 °C. (A) Storage (*G*') and loss (*G*'') moduli for the two different hydrogels measured at a strain amplitude of 1 %. (B) Zoom into the relevant range of *G*' (plotted linearly) to visualize the increase in *G*' more clearly. Differences in *G*' between PIC-A4B4 and PIC-0 at 10^{-3} s^{-1} and 10^{1} s^{-1} are indicated.

To quantify the contribution of the CC crosslinks above (10^{1} s^{-1}) and below (10^{-3} s^{-1}) f_{max}, *G*' was compared for PIC-A4B4 and PIC-0 (Table S10). This comparison shows that the presence of CC-A4B4 crosslinks leads to a 20 % increase in *G*' at frequencies below f_{max}. In contrast, *G*' is increased by 60 % at frequencies above f_{max}. Together, this clearly shows the strong and dynamic contribution of CC-A4B4 to the overall viscoelastic properties of PIC-A4B4.

Supplementary table 10. Contribution of CC-A4B4 to the storage modulus of the hydrophobically bundled PIC hydrogel at different frequencies (55 °C). The datasets indicated in bold are shown in Figure S7. Each measurement was performed in triplicate, using a freshly prepared sample. The values for each sample as well as their mean and standard error of the mean (SEM) are shown.

**Sample**	**PIC-0**		**PIC-A4B4**	
	G' (Pa) - 10^{-3} s^{-1}	G' (Pa) - 10^{1} s^{-1}	G' (Pa) - 10^{-3} s^{-1}	G' (Pa) - 10^{1} s^{-1}
1	**154**	**199**	232	381
2	139	183	212	360
3	230	310	**197**	**360**
**Mean**	**174**	**231**	**214**	**367**
**SEM**	**28**	**40**	**10**	**7**
**Mean increase**	**-**	**-**	**20 %**	**60 %**

8. Comparison of measurement geometries for PEG hydrogels

To validate that the different relaxation times are not a result of the different measurement geometries used (plate-plate for PIC-A4B4 and cone-plate for PEG-A4B4), a control experiment was performed with PEG-A4A4. In this experiment, a plate-plate measurement system and a cone-plate system with the same diameter of 12 mm were compared directly (Figure S8). In the linear viscoelastic range, *G'* lies in a similar range and the yield strain appears to be shifted to slightly lower values when the plate-plate system is used. Most importantly, the crossover between G' and G'' is located at frequencies 1.6-2.2 s^{-1} when measured with the plate-plate system (3.1-4.3 s^{-1} for the cone-plate system). The relaxation time is thus not significantly affected by the measurement system used.

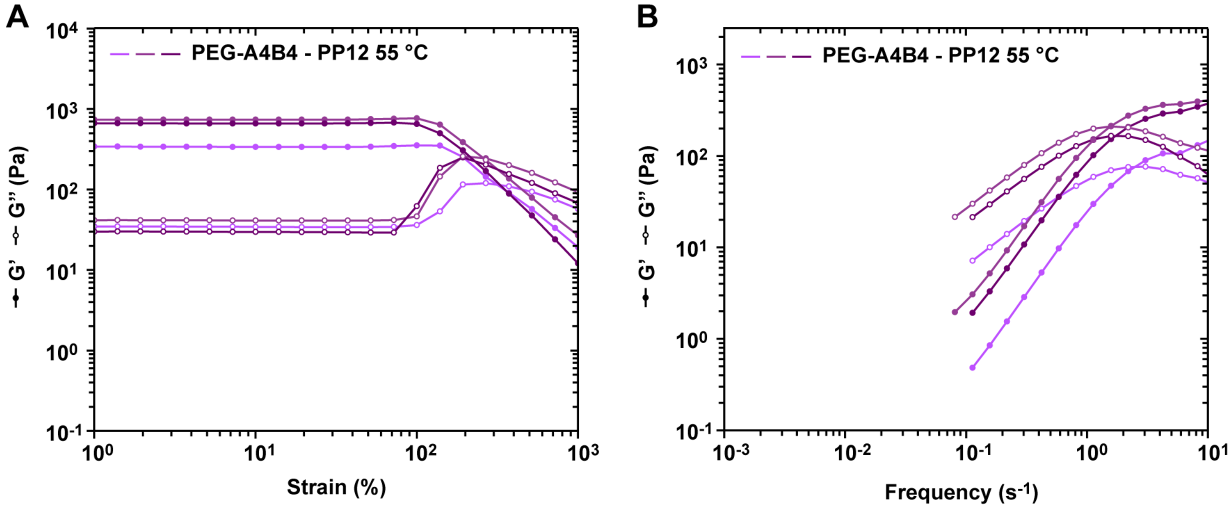

Supplementary figure 8. Properties of PEG-A4B4 hydrogels, measured with a plate-plate system (12 mm diameter, gap 200 μm). (A) Amplitude sweeps performed at a frequency of 1.6 s^{-1} (55 °C). (B) Frequency sweeps recorded at a strain amplitude of 10 % (55 °C). Shown are three independent measurements, each performed with a freshly prepared hydrogel sample. Lines are drawn to guide the eye.
